# The Integration of Artificial Intelligence into Robotic Cancer Surgery: A Systematic Review

**DOI:** 10.3390/jcm14176181

**Published:** 2025-09-01

**Authors:** Agnieszka Leszczyńska, Rafał Obuchowicz, Michał Strzelecki, Michał Seweryn

**Affiliations:** 1 EconMed Europe, Młyńska 9/4, 31-469 Krakow, Poland; agnieszka.leszczynska@autograf.pl (A.L.); misewer@gmail.com (M.S.); 2 Faculty of Medicine, Andrzej Frycz Modrzewski Krakow University, Gustawa Herlinga-Grudzińskiego 1, 30-705 Krakow, Poland; 3 Lux Med Ltd., 02-678 Warsaw, Poland; r.obuchowicz@gmail.com; 4 Institute of Electronics, Lodz University of Technology, 93-590 Lodz, Poland

**Keywords:** artificial intelligence, robotic surgery, cancer surgery, machine learning, intraoperative decision support, surgical automation, AI in oncology, precision surgery

## Abstract

**Background/Objectives**: This systematic review aims to synthesize recent studies on the integration of artificial intelligence (AI) into robotic surgery for oncological patients. It focuses on studies using real patient data and AI tools in robotic oncologic surgery. **Methods**: This systematic review followed PRISMA guidelines to ensure a robust methodology. A comprehensive search was conducted in June 2025 across Embase, Medline, Web of Science, medRxiv, Google Scholar, and IEEE databases, using MeSH terms, relevant keywords, and Boolean logic. Eligible studies were original research articles published in English between 2024 and 2025, focusing on AI applications in robotic cancer surgery using real patient data. Studies were excluded if they were non-peer-reviewed, used synthetic/preclinical data, addressed non-oncologic indications, or explored non-robotic AI applications. This approach ensured the selection of studies with practical clinical relevance. **Results**: The search identified 989 articles, with 17 duplicates removed. After screening, 921 were excluded, and 37 others were eliminated for reasons such as misalignment with inclusion criteria or lack of full text. Ultimately, 14 articles were included, with 8 using a retrospective design and 6 based on prospective data. These included articles that varied significantly in terms of the number of participants, ranging from several dozen to several thousand. These studies explored the application of AI across various stages of robotic oncologic surgery, including preoperative planning, intraoperative support, and postoperative predictions. The quality of 11 included studies was very good and good. **Conclusions**: AI significantly supports robotic oncologic surgery at various stages. In preoperative planning, it helps estimate the risk of conversion from minimally invasive to open colectomy in colon cancer. During surgery, AI enables precise tumor and vascular structure localization, enhancing resection accuracy, preserving healthy tissue, and reducing warm ischemia time. Postoperatively, AI’s flexibility in predicting functional and oncological outcomes through context-specific models demonstrates its value in improving patient care. Due to the relatively small number of cases analyzed, further analysis of the issues presented in this review is necessary.

## 1. Introduction

Since the first iterations of the da Vinci surgical system marked the beginning of computer-assisted surgery, the field has rapidly progressed [1]. Over time, the integration of artificial intelligence (AI) into surgical workflows has intensified, from basic assistive functions to advanced models capable of supporting real-time decision making and personalized surgical strategies.

The application of smart technologies in surgery was already comprehensively addressed in 2018 in a landmark narrative review by Hashimoto et al. [2]. This early work provided a structured overview of how AI could be integrated into various stages of surgical care—ranging from preoperative planning and intraoperative guidance to postoperative monitoring.

In the following years, AI technologies have been continuously refined and expanded. A notable milestone in the knowledge and research on this topic was the systematic review published in 2021 by Moglia et al. [3], which was among the first attempts to summarize the role of AI in robot-assisted surgery. However, most of the included studies were preclinical or based on simulations, focusing primarily on technical validation rather than clinical outcomes. Moglia’s review did not include studies with real oncologic patients and therefore clinical outcomes were largely missing.

The present systematic review offers an updated synthesis of recent studies on the integration of artificial intelligence in robotic surgery, with a specific focus on applications evaluated in real oncological patients. By narrowing the scope to clinically implemented AI tools, this review aims to highlight how these technologies are currently being translated into real-world surgical practice. It examines the use of AI across the perioperative continuum—including preoperative planning and risk stratification, intraoperative decision support and image guidance, as well as postoperative outcome prediction and recovery monitoring—emphasizing its role in enabling personalized oncologic surgery based on individual patient data. Another distinguishing feature of this review is its exclusive focus on studies conducted in populations of oncologic patients. Cancer surgery constitutes one of the most technically and cognitively complex areas of surgical care, due to the need for accurate tumor excision, margin clearance, and preservation of vital structures. Beyond tumor removal, it requires achieving negative margins, performing adequate lymphadenectomy, and preserving vital anatomical and functional structures. These procedures are often carried out in anatomically complex regions and directly affect patient survival and quality of life. Integrating AI into robotic cancer surgery represents a shift from mechanical assistance to intelligent surgical support. AI applications span from preoperative imaging analysis and intraoperative structure recognition to real-time navigation, margin assessment, and outcome prediction. In oncology—where surgical precision is critical—these tools hold particular promise.

To ensure this review’s relevance and timeliness, we included only studies published in the last 18 months. Given the rapid advancements in this field—particularly the emergence of transformer-based models and multi-modal AI architectures—restricting the timeframe to recent years allowed us to capture the most clinically meaningful innovations.

Eligible publications were limited to studies applying AI tools to robotic oncologic surgery based on real patient data with demonstrable clinical potential. We excluded studies using only synthetic or preclinical datasets, non-oncologic indications, or AI applications unrelated to robotic surgery (e.g., conventional laparoscopy or training simulations). This focused approach ensured practical applicability and alignment with the review’s aim of evaluating AI’s real-world contribution to oncologic robotic surgery.

To structure the analysis and facilitate clinical interpretation, the included publications were grouped into three thematic categories: preoperative planning, intraoperative support, and postoperative predictions. This categorization reflects the natural progression of the surgical care continuum and highlights the distinct roles AI can play at each phase—from optimizing risk assessment and surgical strategy before the procedure, through real-time guidance and anatomical recognition during surgery, to forecasting outcomes and informing personalized follow-up after the operation.

While some reviews have examined AI in surgery or robotic systems, the specific intersection of AI, robotics, and oncologic surgery remains underexplored. A dedicated systematic review is therefore both timely and necessary to map current evidence, identify gaps, and inform future clinical and research directions.

## 2. Materials and Methods

### 2.1. Study Design and Search Strategy

This systematic review adhered to the preferred reporting items for systematic reviews and meta-analyses (PRISMA) guidelines to guarantee a thorough and well-organized methodology [4], though the systematic review’s protocol has not been prepared. The search was conducted in June 2025. A literature search was performed across Embase, Medline, Web of Science, medRxiv, Google Scholar, and Institute of Electrical and Electronics Engineers database (IEEE) to ensure comprehensive coverage of both medical and technological aspects of the investigated topic. The search approach combined medical subject headings (MeSH) terms, relevant keywords, and Boolean logic to achieve a wide yet focused selection of studies. The search strings used in each database are presented in Table A1 (Appendix A).

### 2.2. Eligibility Criteria and Study Selection

Studies were eligible for inclusion if they were original research articles published in English between 2024 and 2025, focused on the application of artificial intelligence to any phase of robotic cancer surgery. Only publications applying AI tools to robotic oncologic surgery based on real patient data and demonstrating clinical potential were included. Studies were excluded if they were non-peer-reviewed (e.g., editorials, letters, abstracts, or case reports), used only synthetic or preclinical datasets, addressed non-oncologic indications, or explored AI applications unrelated to robotic surgery (e.g., conventional laparoscopy or surgical training simulations). This focused approach ensured practical applicability and alignment with the review’s aim of evaluating AI’s real-world contribution to oncologic robotic surgery.

**Figure 1:**
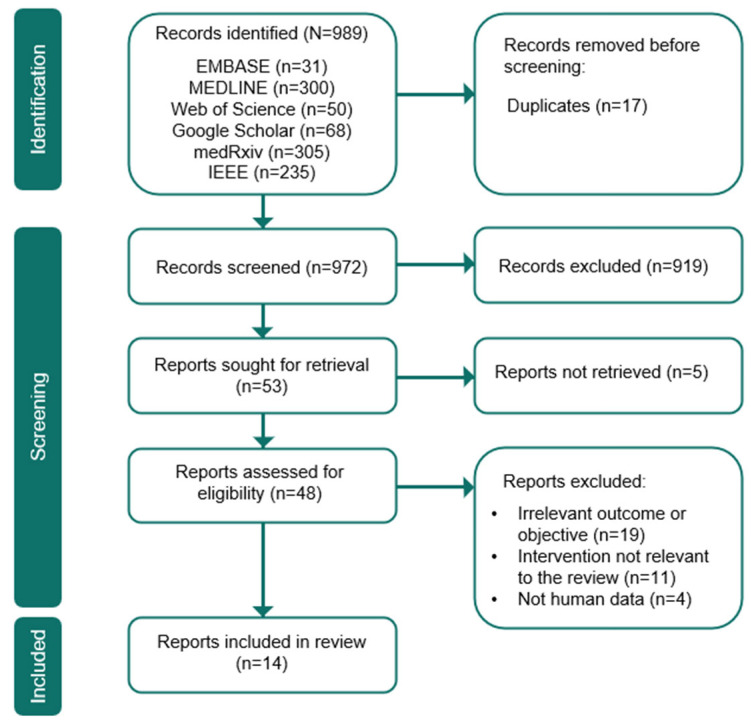
PRISMA flow chart.

The selection of relevant studies was carried out independently and in a blinded manner by two authors (A.L. and M.Se). In case of disagreement, a third author was consulted to reach a consensus. Titles and abstracts were screened for relevance, followed by full-text assessments based on the predefined eligibility criteria. Any discrepancies between the reviewers were resolved through discussion and consensus. The study selection process is summarized in the PRISMA flow diagram, Figure 1.

### 2.3. Data Extraction and Assessment

The following variables were extracted and reported: study characteristics, clinical context, AI application, model evaluation, and clinical relevance. Outcomes were extracted as reported in the studies, without any predefined assumptions regarding which specific results should be collected. This review comprises studies with heterogeneous designs and methodologies, and the endpoints assessed varied substantially. Some studies focused on populations with colorectal cancer, while others examined patients with kidney, prostate, or pancreatic cancer. Additionally, some studies employed a retrospective design, while others were prospective in nature. The outcome measures also differed across studies, with some reporting metrics such as the area under the curve (AUC), while others used the Dice coefficient or Tetrafecta, among other measures. Taken together, these factors made it infeasible to perform a formal meta-analysis. Data were summarized in structured tables by one reviewer and verified by another. A formal quality assessment was conducted by both reviewers using the National Institutes of Health (NIH) criteria and the Transparent Reporting of a multivariable prediction model for Individual Prognosis or Diagnosis–Artificial Intelligence (TRIPOD-AI) guidelines (see Table A2, Appendix A). The collected results were presented in both narrative and tabular form.

## 3. Results

The initial search of the databases identified 989 articles, with 17 duplicates removed. Following a review of titles and abstracts, 921 articles were excluded as they did not meet the inclusion criteria. Of the remaining articles, 51 were deemed potentially relevant and were assessed further; however, 37 of them (as well as the 2 identified during the evaluation) were ultimately excluded due to reasons such as a study objective not aligned with our inclusion criteria [5,6,7,8,9,10,11,12,13,14,15,16,17,18,19,20,21,22,23], an intervention different from the one analyzed [24,25,26,27,28,29,30,31,32,33,34], the study not involving human subjects [35,36,37,38], or unavailability of the full text for our reviewers [39,40,41,42,43]. Ultimately, 14 articles were selected for inclusion in the review [44,45,46,47,48,49,50,51,52,53,54,55,56,57]. Eight studies employed a retrospective design, while six reported data from prospective enrolment. The included studies varied significantly in terms of the number of participants, ranging from 17 in Chen et al. to 26,546 in Emile et al. (Table 1).

### 3.1. Preoperative Planning

The five analyzed studies demonstrate the growing integration of various types of artificial intelligence into preoperative planning in robotic surgery, each using distinct AI approaches to enhance decision making and outcome prediction. Emile et al. [44] developed a logistic regression-based predictive model using the National Cancer Database to estimate the risk of conversion from minimally invasive to open colectomy in colon cancer. Their analysis identified key clinical and tumor-related predictors, and demonstrated that robotic surgery is independently associated with a significantly lower risk of conversion compared with laparoscopy. Huang et al. [45] utilized AI-based analysis of three-dimensional computed tomography (CT) reconstructions of renal tumors to enhance anatomical characterization and improve prediction of Tetrafecta outcomes in robotic-assisted partial nephrectomy, achieving a higher AUC of 0.854 compared with 0.755 obtained with traditional two-dimensional system based on preoperative aspects and dimensions used for an anatomical classification scoring (SPARE). Lu et al. [46] examined how pelvic and prostate anatomical dimensions affect the surgical difficulty of robot-assisted radical prostatectomy by applying an eXtreme Gradient Boosting (XGBoost) model to magnetic resonance imaging (MRI)-derived metrics. The model outperformed logistic regression in predicting prolonged operative time and increased blood loss. Mei et al. [47] developed a convolutional neural network (CNN) trained directly on pelvic MRI images to predict intraoperative difficulty in radical prostatectomy, achieving high predictive performance (AUC ~0.85) and outperforming traditional morphometric models based on manually extracted anatomical features. Finally, Saikali et al. [48] developed artificial neural network models using preoperative clinical and functional variables from over 8500 patients treated at a high-volume prostate cancer referral center, aiming to predict recovery of urinary continence (AUC 0.68) and erectile function (AUC 0.74) one year after robotic-assisted radical prostatectomy.

### 3.2. Intraoperative Support

A synthesis of seven recent studies demonstrates how various AI-based solutions contribute to increasing the precision and safety of surgical procedures. It is important to note that these studies vary in size. For instance, some studies, such as those by Amparore et al. [49] and Chen et al. [53], are early feasibility research with relatively small sample sizes (20 and 17 patients, respectively). While these pilot studies provide valuable insights into the potential of AI in intraoperative support, their results should be interpreted with caution, and further validation in larger populations is needed. In contrast, studies like Bannone et al.’s [50] EX-MACHYNA trial, which includes 169 patients, offer more robust evidence, demonstrating the broader applicability and effectiveness of AI-driven surgical tools across diverse patient groups.

Amparore et al. [49] and Shi et al. [50] investigated the integration of AI with augmented reality (AR) in robot-assisted partial nephrectomy. Both teams developed AI-enhanced systems combining computer vision and machine learning to automatically align preoperative three-dimensional (3D) anatomical models with intraoperative imaging. This enabled accurate localization of tumors and vascular structures, thereby assisting surgeons in performing more precise resections, improving healthy tissue preservation, and reducing warm ischemia time. These findings highlight the value of AI in providing spatial navigation support and reducing cognitive load during complex minimally invasive procedures.

Another promising application of AI in robotic cancer surgery is intraoperative tissue recognition. In the EX-MACHYNA trial, Bannone et al. [51] combined hyperspectral imaging with deep learning to develop an AI-driven system capable of distinguishing malignant from benign tissues during surgery. This approach, termed “surgical optomics,” enabled real-time automatic tissue classification with high diagnostic accuracy and holds potential for supporting intraoperative margin control in oncologic procedures. Similarly, Mannas et al. [52] integrated stimulated Raman histology (SRH) with AI to assess surgical margins during radical prostatectomy. Their system delivered near real-time feedback on residual cancer presence, demonstrating diagnostic performance comparable to standard histopathology and significantly reducing the time between tissue excision and clinical decision making.

Artificial intelligence is also increasingly applied to real-time anatomical structure segmentation during robotic surgery. Chen et al. [53] developed a CNN to identify and delineate the ureters in robot-assisted radical cystectomy. Their fluorescence-like navigation system provided continuous visual feedback, helping reduce the risk of iatrogenic injury. Similarly, Nakamura et al. [54] applied a semantic segmentation model to accurately highlight the pancreas during robot-assisted gastrectomy—supporting surgeons in avoiding unintended damage to this anatomically challenging and poorly visualized organ. These applications demonstrate how AI-based segmentation can enhance intraoperative safety and improve the precision of complex surgical procedures.

Furube et al. [55] addressed the intraoperative challenge of identifying recurrent laryngeal nerves during robot-assisted minimally invasive esophagectomy. They developed and validated an AI model that analyzed endoscopic video in real time to generate visual cues for nerve localization. This system assisted surgeons in preserving nerve integrity and helped prevent complications such as vocal cord paralysis, demonstrating the value of AI in enhancing nerve safety during complex thoracic procedures.

### 3.3. Postoperative Predictions

Both studies by Geitenbeek et al. [56] and Ghaffar et al. [57] illustrate the clinical utility of AI in predicting postoperative outcomes after robotic cancer surgery, despite differing in surgical context, data sources, and clinical endpoints. Ghaffar et al. applied computer vision techniques to intraoperative video recordings from nerve-sparing radical prostatectomy to quantify neurovascular bundle (NVB) retraction. The extracted image-based features, when integrated into machine learning models, significantly improved the prediction of erectile function recovery, with the AUC increasing from 0.78 to 0.83. In contrast, Geitenbeek et al. [56] analyzed structured clinical and pathological data from a large international multicenter cohort of patients undergoing robotic total mesorectal excision (R-TME). Using an XGBoost model, they predicted the risk of local recurrence with an AUC of 0.76, further enhanced by explainable AI techniques: SHapley Additive exPlanation (SHAP) and local interpretable model-agnostic explanations (LIME) that identified key predictors such as metastasis stage, margin status, and postoperative complications. Although both studies were retrospective and lacked external validation, they demonstrate the flexibility of AI in addressing distinct postoperative outcomes—functional and oncological—through context-specific modeling approaches. Taken together, these studies highlight how AI can complementarily leverage different types of data—structured clinical/pathological information and intraoperative video—to provide both clinical and procedural insights in robotic oncologic surgery.

## 4. Discussion

The present review builds upon the thematic foundations established by the systematic review of Moglia et al. [3], titled “A systematic review on artificial intelligence in robot-assisted surgery”, which was among the first efforts to comprehensively summarize the role of AI in this rapidly evolving surgical field. Both reviews highlight the increasing integration of AI technologies—particularly computer vision, machine learning, and deep learning—into robot-assisted surgical workflows and assess how these tools may enhance surgical performance, improve clinical outcomes, and support decision making. However, revisiting this topic was both necessary and timely for several reasons. First, the field has advanced considerably since the publication of the earlier review, with a marked increase in studies reporting clinical applications and translational outcomes. Second, while Moglia et al. laid a valuable foundation, their review primarily focused on preclinical, technical, and simulation-based studies. Many of the included investigations addressed tool tracking, surgical gesture recognition, or system validation in artificial or non-human environments. In contrast, the current review focuses exclusively on the integration of AI in robotic cancer surgery and includes only studies conducted with real patients.

It should be noted that evidence-based advantages of many other robotic procedures in surgical oncology remain unclear [58]. However, the field is advancing rapidly, with ongoing research continuing to push the boundaries of clinical applicability. For instance, Quero et al. [59] provided in 2022 a comprehensive overview of computer-vision and AI applications in colorectal cancer surgery, such as automatic recognition of surgical phases and guidance during com-plex resections, as an early overview of potential intraoperative AI tools. More recently, Chen et al. [53] presented a more advanced stage of development—demonstrating an early yet clinically grounded implementation of this approach using data from real patients undergoing radical cystectomy.

The primary aim of this review was to synthesize available evidence on how AI is applied in clinical oncological settings and to present findings that reflect actual patient outcomes. By narrowing the scope to real-world oncological procedures, this review seeks to bridge the gap between experimental validation and clinical relevance—thereby providing insights directly applicable to surgical practice and future research directions.

The studies analyzed in the preoperative planning section highlight the growing importance of AI in robotic surgery, with applications including structured data modeling, AI-enhanced 3D imaging, and deep learning. Traditional machine learning algorithms have shown strong performance in analyzing structured clinical data, whereas deep learning—especially convolutional neural networks (CNNs)—has demonstrated superiority in processing medical images and predicting procedural complexity. AI-driven 3D imaging tools also facilitate enhanced anatomical visualization and support the development of personalized surgical strategies. Across the reviewed studies, predictive performance ranged from moderate to high (AUCs 0.68–0.85), underlining AI’s potential to optimize surgical planning, improve risk stratification, and enable patient-centered decision making.

Intraoperative support is one of the most promising areas for AI integration in robotic surgery. The included studies describe diverse AI applications—from segmentation of anatomical structures and fluorescence-like navigation to tissue classification and nerve identification—all aimed at enhancing real-time intraoperative decision making. These tools primarily rely on supervised deep learning models trained on large, annotated datasets and have shown promising results in terms of accuracy, sensitivity, and specificity. While most are still in the early phases of clinical validation, their integration into real-time surgical workflows suggests growing feasibility and utility in improving intraoperative awareness, supporting key maneuvers, reducing complication risk, and ultimately improving patient outcomes.

Finally, the studies by Ghaffar et al. [57] and Geitenbeek et al. [56] underscore AI’s growing role in postoperative outcome prediction following robotic oncologic surgery. By using diverse data sources—intraoperative video in one case and structured clinical data in the other—these studies illustrate how tailored AI models may support personalized risk stratification for both functional recovery and oncologic recurrence.

Despite the promising results obtained in this systematic review, several limitations should be considered. The heterogeneity of the study designs and interventions presented in the analyzed studies made it difficult to draw definitive conclusions regarding the optimal use of AI in specific surgical procedures. The included studies were based on diverse oncology patient populations, most of which were small, and there are still relatively few studies employing real patient data and AI tools in robotic oncologic surgery. Many studies, such as those by Amparore et al. [49], Shi et al. [50] and Chen et al. [53] lacked external validation, which limits the generalizability of their findings. Additionally, a number of studies did not make their code or models publicly available (Table A2), raising concerns about reproducibility and transparency—critical aspects in AI research. Another key challenge is the “black-box” nature of many CNN-based models, where the decision-making process is not easily interpretable. This lack of interpretability can pose a barrier to clinical acceptance, as clinicians may be hesitant to rely on AI predictions without understanding the underlying rationale. Overall, while the findings gathered in this review provide useful insights and serve as a valuable reference point, they do not constitute definitive evidence of the benefits of specific AI applications in surgical practice. Further validation in larger multicenter prospective cohorts, open-access models, and the integration of explainable AI techniques remains necessary to ensure that AI-driven surgical tools can be trusted and safely implemented in clinical practice.

## 5. Conclusions

New medical technologies represent a cornerstone of the future of medicine. Robotic surgery has established itself as an integral part of oncological care and is increasingly recognized as a clinical standard for the treatment of various cancers. The integration of AI into robotic surgery holds the promise of further enhancing the efficiency, precision, and overall quality of surgical care.

AI supports surgeons at multiple stages of the clinical pathway: from preoperative planning, through real-time intraoperative assistance, to the prediction of postoperative outcomes. Evidence suggests that its use can improve surgical precision, reduce the risk of complications, and ultimately contribute to better patient outcomes in robotic cancer surgery.

Looking ahead, AI’s potential in robotic oncologic surgery is vast. Emerging trends such as multimodal AI, which integrates imaging, clinical, and genomic data, hold promise for more personalized surgical planning and risk prediction. Future research should focus on optimizing AI algorithms for greater predictive accuracy and treatment personalization, as well as evaluating their effectiveness and efficiency in clinical practice. Integration with emerging technologies, such as augmented reality and advanced robotics, could further revolutionize minimally invasive procedures. International collaborative datasets may help overcome current limitations of small single-center studies, enhancing model generalizability. Finally, ethical and legal considerations remain crucial: the extent to which intraoperative decision making can be safely delegated to AI, and how responsibility is shared between clinicians and AI systems, will require careful evaluation. Addressing these aspects will be key to ensuring safe, effective, and broadly applicable AI-driven surgical interventions in the future.

## Figures and Tables

**Table 1:** Characteristics of research and data.

Study	Study Characteristics	Clinical Context	AI Application	Model Evaluation	Clinical Relevance
**Preoperative planning**					
Emile et al. (2024) [44]	Retrospective case–control; demographic, clinical, surgical data; internal validation (NCDB)	Conversion from MIS to open colectomy; 30-/90-day mortality, LOS, readmission, OS; 26,546 stage I–III colon cancer patients	ChatGPT-generated R code; multivariate logistic regression; OR-based model; VIF; R code available	OR up to 17.8 (high-risk); reduced to 8.9 with robotics; AUC not reported	May assist in surgical planning and platform selection
Huang et al. (2024) [45]	Retrospective cohort; demographic, perioperative, CT imaging; internal only	RAPN; Tetrafecta (WIT < 25 min, negative margins, no major complications, preserved renal function); 141 patients	AI-based segmentation + 3D reconstruction (Yorktal IPS); automated SPARE score + Tetrafecta prediction	AUC: 0.854 (3D) vs. 0.755 (2D); categorical 0.658 vs. 0.643	Improved risk stratification and surgical planning using 3D imaging
Lu et al. * (2024) [46]	Prospective cohort; anatomical (MRI) and surgical data; internal validation	RARP; operative time, EBL, surgical margin; 219 patients	XGBoost; prediction of prolonged operative time; SHAP explainability	XGBoost outperformed logistic regression (no details reported)	Identifying challenging anatomy may aid surgical planning
Mei et al. (2025) [47]	Retrospective DL with segmentation; MRI + spatial features; internal and external validation	Surgical difficulty in RARP; EBL and OT; 290 patients with MRI	nnUNet_v2 + modified PointNet; regression of spatial metrics	Dice = 0.8641 (segmentation); mm-level landmark accuracy	New evaluation scheme for preoperative planning
Saikali et al. (2025) [48]	Retrospective observational (single center); preoperative clinical data; internal comparison only	Prediction of urinary continence and erectile function at 12 months post-RARP; 8524 patients	ANN; prediction of continence and potency; feature importance analysis	AUC: 0.68 (continence), 0.74 (potency)	Patient counseling and care optimization
**Intraoperative support**					
Amparore et al. (2024) [49]	Prospective single-center; intraoperative video + clinical data; internal validation only	Robotic nephrectomy; overlay time and procedure safety; 20 patients with renal masses	Computer vision + CNN; automatic 3D model registration; expert visual assessment	Overlay time: CV~7 s, CNN~11 s	Faster accurate AR-assisted surgery
Shi et al. (2025) [50]	Prospective–retrospective development; preop CT + laparoscopy video; clinical use only	MIPN patients; navigation and dissection standardization; 46 patients	Augmented reality with AI overlay; real-time anatomic guidance; 3D visual overlay	Performance not quantified	Improved surgical precision and consistency
Bannone et al. (2024) [51]	Prospective multicenter; hyperspectral + RGB images; internal + external (inter-center)	Tissue recognition during surgery; 13 tissue classes; 169 patients	CNN; real-time tissue segmentation; expert review only	TPR: skin 100%, liver 97%; Dice > 80%	Improved intraoperative tissue identification
Mannas et al. (2024) [52]	Prospective pilot; 121 intraoperative SRH images; tested on 10 patients	Surgical margin interpretation in RALP; accuracy, sensitivity, specificity vs. pathology; 22 patients	CNN; classify margin status in SRH; no internal explainability methods	Accuracy 98%, sensitivity 83%, specificity 99% (surgeons)	Supports intraoperative decision making; may reduce positive margins
Chen et al. (2025) [53]	Prospective developmental; 730 RGB images from RARC; retrospective validation on 41 images	Real-time ureter segmentation during RARC; segmentation quality (Dice, IoU, recall, precision); 17 cases	CNN; semantic segmentation of ureter; limited explainability (surgeon only)	Dice 0.71; IoU 0.55; recall 0.90; precision 0.60	Reduces ureter misidentification; improves safety and training
Nakamura et al. (2024) [54]	Retrospective image-based; annotated surgical video frames; internal test set	Robot-assisted gastrectomy; pancreas localization; 926 train, 232 val., 80 test images; 10 surgeons	Semantic segmentation (HRNet); visual overlay (mask)	Precision 0.70, recall 0.59, Dice 0.61	May improve anatomy recognition intraoperatively; reduce POPF
Furube et al. (2024) [55]	Retrospective multicenter; surgical videos from RAMIE; external validation (8 videos)	Intraoperative RLN identification; IoU, recognition rate improvement; 128 surgeries	Deep learning (CNN assumed); semantic segmentation and localization of RLN	IoU: 0.40 (right), 0.34 (left); accuracy increased from 46.9% to 81.3%	May improve nerve identification and reduce complications
**Postoperative predictions**					
Geitenbeek et al. (2025) [56]	Retrospective multicenter cohort; clinical/pathological data; internal cross-validation	Local recurrence after R-TME; 3-year LR (3.8%) prediction; 1039 rectal cancer patients in 6 EU countries	ML (XGBoost, others); SHAP for feature importance; LR prediction	XGBoost: accuracy 77.1%, AUC 0.76	Supports safe R-TME; helps identify patients at high LR risk
Ghaffar et al. (2025) [57]	Retrospective video-based cohort; surgical video + clinical data; 4 centers	Nerve-sparing technique vs. erectile recovery; AUC for 12 mo erectile function; 64 patients, 1104 NVB retractions	Computer vision + supervised ML (RF, MLP, XGBoost); gesture-derived visual features	RF: AUC 0.83; MLP: AUC 0.74; XGBoost: AUC 0.78; 5-fold nested CV	Real-time alerts; ICC 0.68–0.76; potential training tool for surgeons

* Only original data. 3D: three-dimensional; AI: artificial intelligence; ANN: artificial neural network; AR: augmented reality; AUC: area under the curve; ChatGPT: chat generative pre-trained transformer; CNN: convolutional neural network; CT: computed tomography; CV: computer vision; DL: deep learning; EBL: estimated blood loss; HRNet: high-resolution network; ICC: intra-class correlation coefficient; IoU: intersection over union; IPS: image processing system; LOS: length of stay; LR: local recurrence; MIPN: minimally invasive partial nephrectomy; MIS: minimally invasive surgery; ML: machine learning; MLP: multi-layer perceptron; MRI: magnetic resonance imaging; NCDB: the National Cancer Database; NVB: neurovascular bundle; OR: odds ratio; OS: overall survival; OT: operation time; POPF: postoperative pancreatic fistulas; RALP: robotic-assisted laparoscopic radical prostatectomy; RAMIE: robot-assisted minimally invasive esophagectomy; RAPN: robot-assisted partial nephrectomy; RARC: robot-assisted radical cystectomy; RARP: robot-assisted radical prostatectomy; RF: random forest; RGB: red green blue; RLN: recurrent laryngeal nerve; R-TME: robot-assisted total mesorectal excision; SHAP: SHapley Additive exPlanation; SPARE: scoring system based on preoperative aspects and dimensions used for an anatomical classification; SRH: stimulated Raman histology; Tetrafecta: optimal perioperative outcomes in nephron-sparing surgery; TPR: true positive rate; VIF: variance inflation factor; WIT: warm ischemic time; XGBoost: eXtreme Gradient Boosting.

## Appendix A

**Table A1:** Search strings used across databases for the systematic literature review.

Database	Search String
EMBASE	((((((((surgical AND ‘procedure’/exp OR surgical) AND oncology OR oncologic) AND surgery OR cancer) AND surgery OR tumor) AND resection OR oncological) AND resection OR tumor) AND removal OR cancer) AND operation*) AND (‘neoplasm’/exp OR cancer OR tumor OR tumour OR malignan* OR oncology OR neoplasm*) AND ((((((‘robotics’/exp OR robot) AND assisted AND ‘surgery’/exp OR robotic) AND surgery OR ‘robot assisted’) AND surgery OR surgical) AND robot* OR robotic) AND system*) AND (((((((artificial AND ‘intelligence’/exp OR machine) AND ‘learning’/exp OR deep) AND learning OR neural) AND network* OR predictive) AND algorithm* OR artificial) AND intelligence OR machine) AND learning OR ai))
MEDLINE	((exp Artificial Intelligence/) OR (exp Machine Learning/) OR (exp Neural Networks, Computer/) OR (artificial intelligence or AI or machine learning or deep learning or neural network* or predictive algorithm*).mp.)) AND ((exp Robotic Surgical Procedures/) OR (robotic surgery or robot-assisted surgery or surgical robot* or robotic system*).mp. OR (exp Robotics/)) AND ((exp Neoplasms/) OR (cancer or oncology or tumour or tumor or neoplasm* or malignan*).mp.) AND ((exp Surgical Procedures, Operative/) OR (oncologic surgery or surgical oncology or cancer surgery or tumor resection or oncological resection or tumor removal).mp.)
Web of Science	Refine results for TS=(“artificial intelligence” OR “machine learning” OR “deep learning” OR “neural network*” OR “predictive algorithm*” OR “AI”) AND TS=(“robotic surgery” OR “robot-assisted surgery” OR “surgical robotics” OR “robotic system*” OR “surgical robot*”) AND TS=(“cancer” OR “oncology” OR “tumor” OR “tumour” OR “neoplasm*” OR “malignan*”) AND TS=(“surgery” OR “surgical procedure” OR “oncologic surgery” OR “cancer surgery” OR “complex surgery” OR “tumor resection” OR “tumour removal”) and 2024 or 2025 (Publication Years) and Early Access or Review Article or Article (Document Types) and English (Languages)
Google Scholar	(“artificial intelligence” OR “machine learning” OR “deep learning” OR “neural networks”) AND (“robotic surgery” OR “robot-assisted surgery” OR “surgical robotics”) AND (“oncology” OR “cancer” OR “tumor” OR “tumour” OR “neoplasm”) AND (“complex surgery” OR “oncologic surgery”)
medRxiv	(“artificial intelligence” OR “machine learning” OR “deep learning”) AND (“robotic surgery” OR “robot-assisted surgery” OR “surgical robotics”) AND (“oncology” OR “cancer” OR “neoplasm”) AND (“complex surgery” OR “tumor resection” OR “oncologic surgery”)
IEEE	(“Full Text Only”:robotic surgery OR “Full Text Only”:surgical robotics) AND (Full Text Only”:oncology OR “Full Text Only”:cancer OR “Full Text Only”:tumor) AND (Full Text Only”:artificial intelligence OR “Full Text Only”:deep learning)

**Table A2:** Quality assessment of included publications.

Author (Year)	TRIPOD-AI Score * Assessment and Comment		NIH Assessment ** Score and Comment		Overall *** Assessment
Mannas et al. (2025) [52]	Good	Very well-prepared publication, strong validation and results, no model release.	Good	Well-executed with reliable data sources and outcome evaluation.	3
Mei et al. (2025) [47]	Good	Full documentation, interpretability, multicenter validation, code available.	Good	Innovative approach with appropriate use of AI. Needs external validation and clearer reporting.	3
Amparore et al. (2024) [49]	Fair+	Very well described methodology, detailed description of pipeline and metrics; no code and external validation	Good	Comprehensive design with clearly defined exposure and outcome measures.	2
Bannone et al. (2024) [51]	Fair+	Full architecture and metrics; no model release and limited interpretability.	Good	Clear methodology with a focus on practical implementation.	2
Chen et al. (2025) [53]	Fair+	Good methodology and clinical application, no interpretability and code.	Good	Real-world clinical data supports robustness of early clinical use.	2
Emile et al. (2024) [44]	Fair+	Strong clinical analysis, multicenter; however, full interpretability and model availability are lacking.	Good	Well-designed with clearly defined objectives and consistent methodology. Limited external validation.	2
Furube et al. (2024) [55]	Fair+	Model works intraoperatively, good methodology; no external validation and code.	Good	Innovative AI use with clear patient segmentation.	2
Geitenbeek et al. (2025) [56]	Fair+	Extensive clinical analysis, good metrics; no interpretability and model.	Good	Comprehensive design with appropriate outcome tracking.	2
Lu et al. (2024) [46]	Fair+	Real-time system, but no interpretability and code, internal validation only.	Good	Robust statistical methods and adequate sample size. Some missing details in handling confounders.	2
Saikali et al. (2025) [48]	Fair+	Good implementation and analysis, no code and external validation.	Good	Thorough methodology and strong outcome focus. Reporting of model performance could be expanded.	2
Shi et al. (2025) [50]	Fair+	Good presentation of results, but no code and external validation.	Good	Strong performance metrics with appropriate AI integration.	2
Ghaffar et al. (2025) [57]	Fair	Innovative topic but missing many key elements of AI reporting.	Fair	Lacks confounder control and statistical depth, but relevant AI usage.	1
Huang et al. (2025) [45]	Good	Highest level of detail, multicenter validation, partially available model.	Fair	Good clinical relevance but lacks transparency in reporting and has potential selection bias.	1
Nakamura et al. (2024) [54]	Fair	No interpretability and code; limited description of the model architecture.	Good	Strong methodology with robust outcome definitions.	1

* TRIPOD-AI scale: good 15–17; fair+ 12–14.5; fair 10–11.5. ** NIH scale: good—the study fulfills most key criteria. There are no major methodological flaws, and the risk of bias is low. The results are considered reliable and valid; fair—the study meets some criteria but has some methodological weaknesses, such as incomplete blinding, limited data reporting, or unclear control of confounding. These do not critically undermine the results; poor—the study has major limitations, such as unclear population, lack of temporality between exposure and outcome, uncontrolled confounding, or substantial loss to follow-up. There is a high risk of bias, and the results are likely unreliable. *** 3—good and good; 2—fair+ and good; 1—good/fair and fair; 0—fair/poor and poor. NIH: National Institutes of Health; TRIPOD-AI: Transparent Reporting of a multivariable pre-diction model for Individual Prognosis or Diagnosis–Artificial Intelligence.
